# Multimorbidity patterns and disability and healthcare use in Europe: do the associations change with the regional socioeconomic status?

**DOI:** 10.1007/s10433-023-00795-6

**Published:** 2024-01-03

**Authors:** Lluís Zacarías-Pons, Oriol Turró-Garriga, Marc Saez, Josep Garre-Olmo

**Affiliations:** 1 Research Group on Aging, Disability and Health, Girona Biomedical Research Institute (IDIBGI), Girona, Catalonia Spain; 2 Glòria Compte Research Institute, Fundació Salut Empordà, Figueres, Girona, Spain; 3 Research Group on Statistics, Econometrics and Health (GRECS), University of Girona, Girona, Spain; 4 CIBER of Epidemiology and Public Health (CIBERESP), Madrid, Spain; 5 Serra-Húnter Professor, Department of Nursing, University of Girona, Girona, Spain

**Keywords:** Multimorbidity, Health inequities, Quality of life, Aged, Latent class analysis

## Abstract

Multimorbidity, the concurrence of several chronic conditions, is a rising concern that increases the years lived with disability and poses a burden on healthcare systems. Little is known on how it interacts with socioeconomic deprivation, previously associated with poor health-related outcomes. We aimed to characterize the association between multimorbidity and these outcomes and how this relationship may change with socioeconomic development of regions. 55,915 individuals interviewed in 2017 were drawn from the Survey of Health, Ageing and Retirement in Europe, a population-based study. A Latent Class Analysis was conducted to fit multimorbidity patterns based on 16 self-reported conditions. Physical limitation, quality-of-life and healthcare utilization outcomes were regressed on those patterns adjusting for additional covariates. Those analyses were then extended to assess whether such associations varied with the region socioeconomic status. We identified six different patterns, labelled according to their more predominant chronic conditions. After the “healthy” class, the “metabolic” and the “osteoarticular” classes had the best outcomes involving limitations and the lowest healthcare utilization. The “neuro-affective-ulcer” and the “several conditions” classes yielded the highest probabilities of physical limitation, whereas the “cardiovascular” group had the highest probability of hospitalization. The association of multimorbidity over physical limitations appeared to be stronger when living in a deprived region, especially for metabolic and osteoarticular conditions, whereas no major effect differences were found for healthcare use. Multimorbidity groups do differentiate in terms of limitation and healthcare utilization. Such differences are exacerbated with socioeconomic inequities between regions even within Europe.

**Supplementary Information:**

The online version contains supplementary material available at 10.1007/s10433-023-00795-6.

## Introduction

Multimorbidity, the presence of several chronic conditions, is a rising concern that is continuously increasing the years lived with disability, according to the 2017 Global Burden of Disease Study (James et al. 2018). Since multimorbidity is related to an increase in healthcare utilization (Frølich et al. 2019; Chen et al. 2018), it poses a major burden on healthcare systems worldwide (James et al. 2018; Picco et al. 2016), especially in aging populations. Some studies also documented an association between multimorbidity and quality-of-life-related variables, such as grip strength (Yorke et al. 2017) and disability (Qian and Ren 2016).

Recently, several publications have used latent class analysis (LCA) to fit several multimorbidity patterns and characterize their association with healthcare expenditure (Bayes-Marin et al. 2020; Juul-Larsen et al. 2020; Olaya et al. 2017; Whitson et al. 2016; Tromp et al. 2018) and quality of life (QoL) and functionality measures (Olaya et al. 2017; Larsen et al. 2017; Park et al. 2019; Zheng et al. 2020). This methodology allows a person-centered approach, in which individuals are grouped into different classes with different probabilities of having several chronic conditions. Health outcomes are then regressed on class membership to provide additional information about their severity, generating evidence that may be useful for guiding health promotion strategies.

In addition, recent publications reported a relationship between a low socioeconomic status and multimorbidity, frailty and disability (Dugravot et al. 2020), and between belonging to a low socioeconomic group and living in an economically deprived area and a higher healthcare expenditure, even after adjusting for multimorbidity (Zhao et al. 2020). However, little is known about how the association of multimorbidity with healthcare expenditure and physical limitations varies with regional economic deprivation. Stratifying by region socioeconomic status when analyzing the relationship between multimorbidity patterns and outcomes may shed light on this interaction.

In this study, our aim was to examine the association between multimorbidity patterns and healthcare utilization, physical limitations and QoL in a representative sample of Europeans aged 50 years and older. Additionally, we aimed to investigate whether this association was modified with the socioeconomic status of the region.

## Methods

### Data sources

The individual data used in this study were obtained from the Survey of Health Ageing and Retirement in Europe (SHARE), a multidisciplinary interview-based study which collects health, socioeconomic and social network data of people aged 50 and older (Börsch-Supan et al. 2013). We analyzed information from the 7th wave of data collection, performed in 2017 (Börsch-Supan 2019), because it is the SHARE’s wave embracing the largest amount of different European countries. These 26 states are Austria, Belgium, Bulgaria, Croatia, Cyprus, Czech Republic, Denmark, Estonia, Finland, France, Germany, Greece, Hungary, Italy, Latvia, Lithuania, Luxembourg, Malta, Poland, Portugal, Romania, Slovakia, Slovenia, Spain, Sweden and Switzerland.

The SHARE dataset also contains, for the baseline interview, the location of the surveyed households according to the Nomenclature of Territorial Units for Statistics (NUTS) classification system, mainly for weighting purposes. In our study, we used this valuable information to assign the socioeconomic status to each individual’s region. The socioeconomic status of the included NUTS regions was retrieved from Eurostat. The chosen indicator was the gross domestic product (GDP) in purchasing power (PPS) per inhabitant by NUTS 2 region in 2017 (European Commission 2017), the year in which the 7th wave of SHARE was conducted. Since data regarding Switzerland’s socioeconomic status by region were not available in this dataset, we excluded this country from our analyses.

### Variables

#### Chronic conditions

Multimorbidity patterns were based on the 16 self-reported chronic conditions that were probed during the 7th wave of SHARE interviews. The presence of 15 of these conditions (heart attack, hypertension, hypercholesterolemia, stroke or cerebral vascular disease, diabetes or high blood sugar, chronic lung disease (COPD), cancer, stomach or duodenal ulcer, Parkinson disease, cataracts, dementia, other affective or emotional disorders, rheumatoid arthritis, osteoarthritis and chronic kidney disease) was assessed using the following question: “Has a doctor ever told you that you had/Do you currently have any of the conditions on this card?”. Additionally, self-referred osteoporosis treatment was included as a proxy for this condition asked with another query: “Our next question is about the medication you may be taking. Please look at card 8. Do you currently take drugs at least once a week for problems mentioned on this card?”.

#### Outcomes

To assess the relationship between multimorbidity patterns and healthcare use, physical limitation, and QoL, we included several indicators probed during SHARE interviews. These variables were included in the analysis as binary or continuous.

The variables addressing physical limitations and coded as binary consisted in (1) having one or more activity daily limitations (ADL) (Katz 1963), (2) having one or more instrumental activity daily limitations (IADL) (Lawton and Brody 1969), (3) being limited according to the global activity limitation index (GALI) (Robine et al. 2003) and (4) having two or more mobility, arm function and fine motor limitations. Self-perceived health (Ware and Gandek 1998) was also included as a binary variable, coding for a “poor,” “fair” or “good” self-reported health against the “very good” and “excellent” categories. The score of the 12-item version of the control, autonomy, self-realization and pleasure (CASP) scale (Hyde et al. 2003) was analyzed as a continuous variable to assess quality of life, ranging from 0 (bad QoL) to 12 (good QoL). The maximum handgrip strength (measured using a manometer during SHARE interviews) was also included as a continuous variable in our analyses to assess physical limitation, since it has been previously reported as a powerful biomarker of aging and “vital sign” of health (McGrath et al. 2020). The number of doctor visits (binary, categorized as “3 or less” or “4 or more”) and being hospitalized or not (binary) during the year preceding the interview were the measures included to assess the relation between the multimorbidity patterns and healthcare use.

#### Covariates

Several additional variables were included to adjust the association between multimorbidity patterns and the outcomes, that might have been confounded by other covariates with a different distribution across multimorbidity groups and that might be in turn related to the modeled outcomes. To account for physical characteristics, age was included as a continuous variable, sex was included as a binary covariate and the body mass index (BMI) was operationalized as a categorical variable, coding for being overweight and obese according to WHO’s BMI threshold values (WHO 2020).

Furthermore, the individual socioeconomic level was also considered in our analyses including two additional covariates: the educational level and the self-reported ability to make ends meet considering their monthly income. The former was categorized into “Pre-secondary education,” “Secondary education” and “Post-secondary education.” The latter had four categories: “With great difficulty,” “With some difficulty,” “Fairly easily” and “Easily.”

European regions were classified according to their socioeconomic status. Since NUTS information is registered only at the baseline interview and this regional classification system has been constantly changing, we had to converse the regional codes from one version to the subsequent from 2003 to 2016. Further detailed information on these conversions is available in Supplementary Materials (Additional file 1). We used the 2nd NUTS classification level except for participants from Germany (having codes only for the 1st level) and from the Polish *PL9* region (also considered using the 1st level region).

**Fig. 1:**
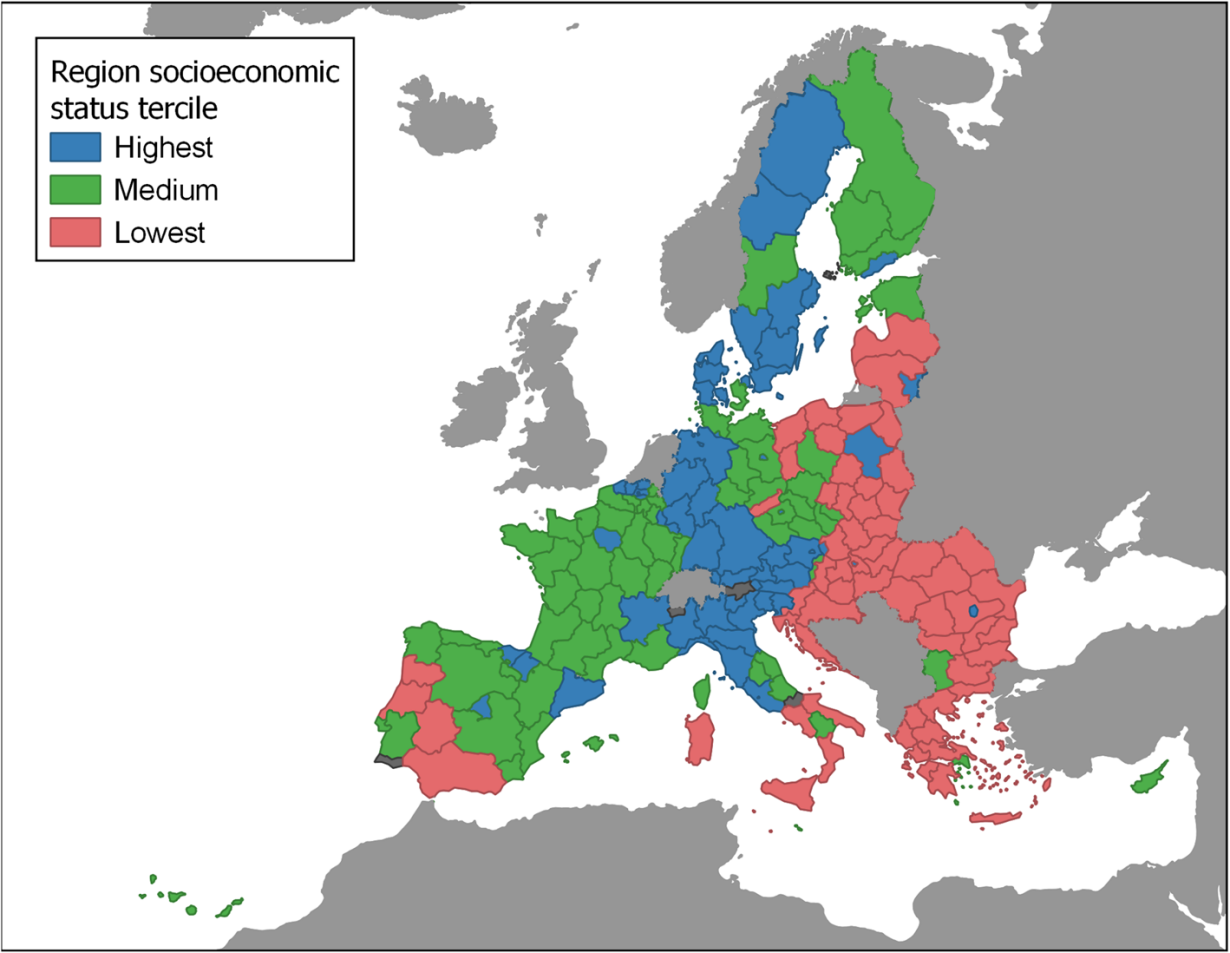
Socioeconomic status tercile for each NUTS 2 region. Regions that met the inclusion criteria but did not have any participant are colored in dark gray

We categorized the continuous region socioeconomic status measure into terciles after assigning this variable at the individual level. The final classification of each region is shown in the map displayed in Fig. 1.

### Statistical analyses

In this study, LCA was the chosen technique to fit multimorbidity patterns. In such approach, a defined number of classes is fit with class-specific probabilities of having each included chronic condition. Within the same model, class membership is estimated for each individual (Collins and Lanza 2009). In order to determine the optimal number of classes, several LCA models up to 8 classes were estimated and then compared using different fit indicators: the Akaike information criterion (AIC), the Bayesian information criterion (BIC), the sample-size adjusted BIC (aBIC) and the Lo–Mendell–Rubin likelihood ratio test (LMR) (Nylund et al. 2007). In order to check whether the conformation of latent classes might be affected by the survey sampling, a sensitivity analysis was carried out by fitting the chosen solution adding weights according to each country population size.

Once the multimorbidity classes were identified, the analysis of the association of class membership with each analyzed outcome was performed using the Bolck–Croon–Hagenaars (BCH) method that does not change the latent class-specific disease probabilities (Asparouhov and Muthén 2020). Such method uses weights to reflect the measurement error of the classification of individuals within latent classes, and it has a better performance than other methods when modeling continuous distal outcomes whose variance might be unequal across latent classes (Asparouhov and Muthén 2020). This approach allowed to adjust for sex, age, BMI, education level, ease to make ends needs and regional socioeconomic level tercile group, including these variables as covariates of each outcome when estimating class-specific probabilities (for categorical binary outcomes) and means (for continuous outcomes).

Our study also aimed to determine how the multimorbidity association with physical limitation, QoL and healthcare use might vary with the region socioeconomic status. To that end, the previous BCH models were extended to stratify the latent class membership effects over the outcomes by region socioeconomic status terciles, as represented in Additional file 1. Fig. S1. Further detailed information on how these models were performed is available in Supplementary Methods 2. To validate our results, additional sensitivity analyses including the region socioeconomic status as a continuous variable were conducted.

Results are expressed as absolute numbers and percentages, mean, standard deviation (SD), odds ratios and adjusted mean differences and ratios of odds ratios (for mediation effects) and 95% CIs. All statistical analyses were conducted using R and MPlus 8.4. We employed an alpha level for statistical significance of 0.05 (two-tailed).

## Results

The final sample size included in the analyses after applying exclusion criteria (Additional file 1  Fig. S2) consisted in 55,915 individuals. The mean age was 67.24 (SD = 9.28), and the 55.76% were females. Other descriptive statistics are reported in Additional file 1 Table S1.

**Fig. 2:**
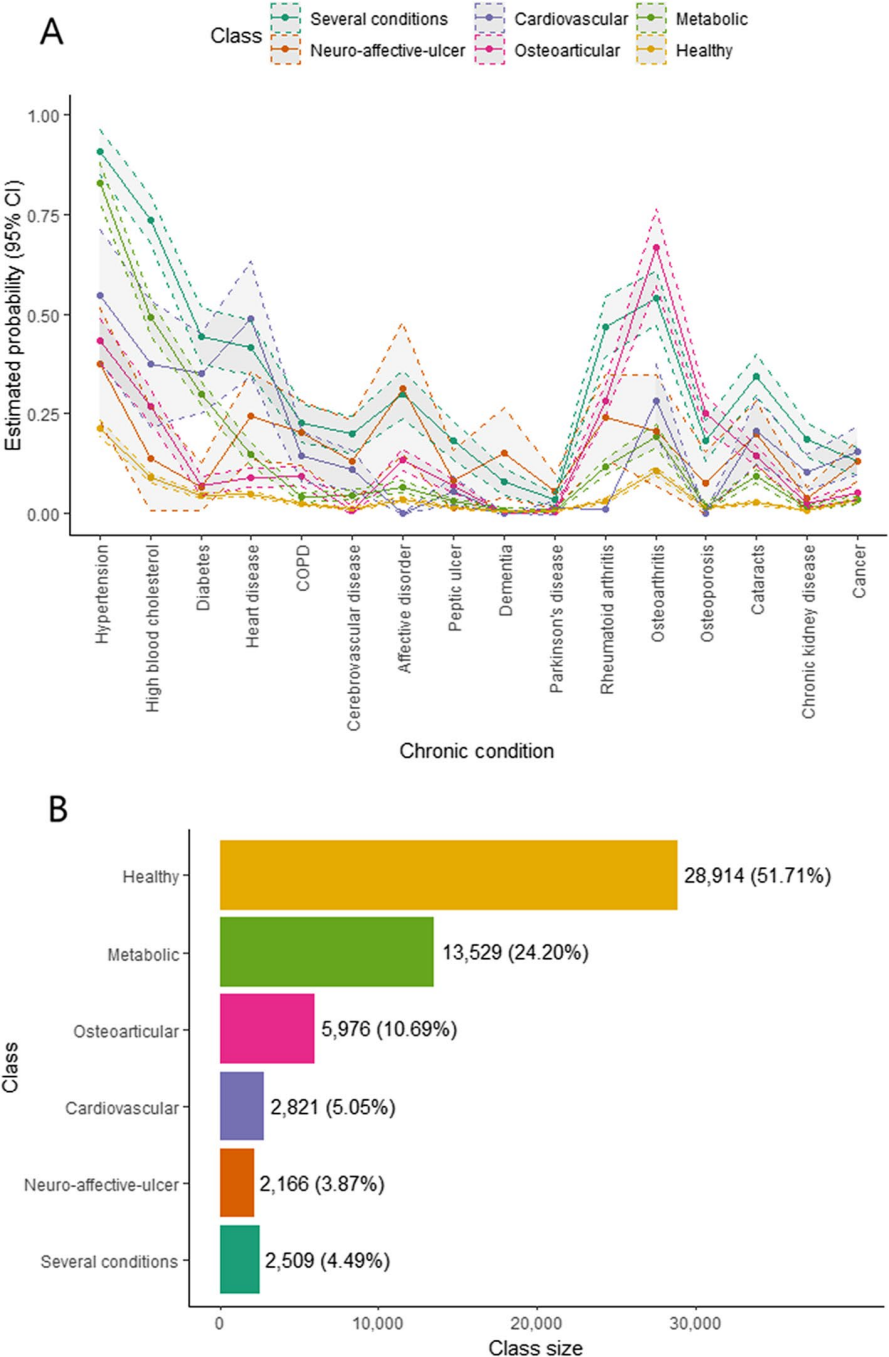
**A** Class-specific probabilities of having each chronic condition and **B** Class sizes based on estimated membership probabilities

The chosen class number for the subsequent analyses was six. The followed selection procedure is reported in Supplementary Results. The class-specific chronic condition probabilities for the 6-class model are displayed in Fig. 2A and Additional file 1 Table S3. Class classification results are shown in Fig. 2B. Classes were named according to their most common chronic conditions. The class with low probabilities for every chronic condition was labelled “Healthy.” The class with high probability of having hypertension, high blood cholesterol and diabetes was labeled “Metabolic,” and the class with high probability of osteoarthritis, rheumatoid arthritis and osteoporosis was named “Osteoarticular.” The class with high probability of heart disease and cerebrovascular disease was named “Cardiovascular,” and the class with high probability of affective disorders, peptic ulcer, dementia and cerebrovascular disease was labeled “Neuro-affective-ulcer.” The LCA yielded a class with the highest class-specific probabilities for every chronic condition, labeled as “Several conditions.” The sensitivity analysis using sampling weights yielded a similar composition of the latent classes (Additional file 1 Tables S3 and S4).

### Outcomes

**Fig. 3:**
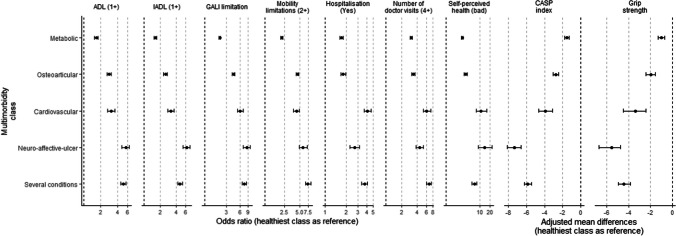
Outcome differences between each multimorbid class and the healthiest class (set as the reference). The estimated models were adjusted for sex, age, BMI, education level and ease to make ends needs. The first six outcomes were analyzed as categorical (odds ratios), and the last three were included as continuous (adjusted mean differences)

The results regarding the association of latent class membership with the studied outcomes adjusted for covariates are displayed in Fig. 3 and Additional file 1 Table S6. The unadjusted associations are displayed in Additional file 1 Table S5. The “healthy” class was set as the reference when computing odds ratios and mean differences.

Regarding the four indicators involving physical limitations, all the non-healthy classes had an increased risk of being limited. The “metabolic” class had the lowest odds ratios for having one or more ADL and IADL limitations, being limited according to the GALI and having two or more mobility limitations. The “osteoarticular” and the “cardiovascular” classes yielded similar risks for the ADL and the mobility limitations, but the latter had larger odds ratios for the IADL and the GALI. The “neuro-affective-ulcer” and the “several conditions” classes showed similar estimates and the largest risks for these limitations.

Belonging to any non-healthy class was related a worse self-perceived health, with the “metabolic” and the “osteoarticular” classes having lower odds ratios than the “several conditions,” the “cardiovascular” and the “neuro-affective-ulcer” groups. Regarding the CASP QoL index, the “metabolic” class had the smallest score reduction compared to the “healthy” class, followed by the “osteoarticular,” the “cardiovascular,” the “several conditions” and the “neuro-affective-ulcer” classes. The grip strength was also lower among non-healthy classes. The “metabolic” class had the smallest reduction, followed by the “osteoarticular” class and by the “cardiovascular,” the “several conditions” and the “neuro-affective-ulcer” classes.

Regarding healthcare use, the odds ratios for having been hospitalized in the last year were similar between the “metabolic” and the “osteoarticular” classes, followed by the “neuro-affective-ulcer” class and by the “several conditions” and the “cardiovascular” classes. The odds ratios of having attended to the doctor visits more than four times in a year (with the “healthy” class as the reference) were similar between the “metabolic” and “osteoarticular” classes and between the “cardiovascular,” the “neuro-affective-ulcer” and the “several conditions” classes.

### Region socioeconomic status interaction

**Fig. 4:**
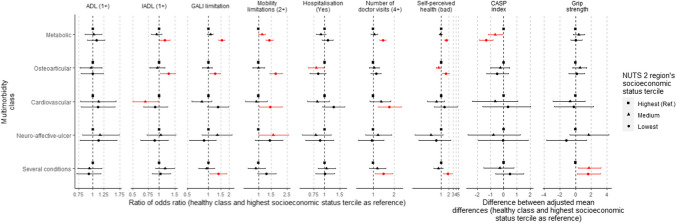
Comparison of the effects of belonging to each multimorbid pattern on health-related outcomes between region socioeconomic status terciles (highest set as reference). Statistically significant estimates are colored in red. The estimated models were adjusted for sex, age, BMI, education level and ease to make ends needs. The first six outcomes were analyzed as categorical (ratio of odds ratios) and the last three were included as continuous (difference between adjusted mean differences)

Figure 4 summarizes how odds ratios and adjusted mean differences within multimorbidity classes varied across region socioeconomic status terciles. The estimates displayed in this figure represent how the several associations of belonging to each multimorbidity class with each outcome within regions in the lowest and medium terciles of socioeconomic status vary in relation to the same associations within the high-socioeconomic status zones.

We found a significantly different association of belonging to the “metabolic” or to the “osteoarticular” class with the probability of having one or more IADL limitations, with an increased odds ratio for those individuals living in a region within the lowest socioeconomic status tercile. The association with the “metabolic” class membership was 1.15 times higher (95% CI = 1.00–1.33) in the most deprived regions compared to the same relationship within the high-socioeconomic status zones. The association of belonging to the “osteoarticular” class was 1.27 times higher (95% CI = 1.03–1.53) for the poorest regions. The odds ratio for the “cardiovascular” class was significantly lower for those individuals within the medium socioeconomic status tercile (0.70 times, 95% CI = 0.50–0.97).

There were also significant differences regarding the association of some classes with the probability of being limited according to the GALI across region socioeconomic status terciles. The odds ratios for the “metabolic” (1.55 times higher, 95% CI = 1.40–1.71), “osteoarticular” (1.25 times higher, 95% CI = 1.04–1.48) and “several conditions” (1.40 times higher, 95% CI = 1.04–1.84) classes increased for the lowest socioeconomic status region tercile.

The association of having two or more mobility limitations and belonging to the “metabolic” or to the “osteoarticular” classes was significantly stronger for individuals living in a region within the lowest socioeconomic status tercile. Within these regions, the association with belonging to the “metabolic” class was 1.41 times stronger (95% CI = 1.26–1.59), the association with being classified in the “osteoarticular” class was 1.72 times stronger (95% CI = 1.43–2.13), and 1.46 (95% CI = 1.02–2.14) with belonging to the “cardiovascular” class.

Regarding self-perceived health, we found increased odds ratios of reporting a worse health status for the “metabolic” (1.59 times higher, 95% CI = 1.35–1.88), “osteoarticular” (1.56, 95% CI = 1.14–2.12) and “several conditions” (1.81, 95% CI = 1.23–2.71) classes within the poorest regions. However, the odds ratio for the “osteoarticular” class within the medium tercile was lower in comparison with the highest (0.78, 95% CI = 0.62–0.99).

The relationship of belonging to the “metabolic” with the CASP QoL score also appeared to differ significantly between region socioeconomic status terciles. The adjusted mean reduction for this class was 1.31 points greater (95% CI = -1.83, − 0.79) within the lowest socioeconomic status tercile and 0.63 points greater (95% CI = -1.18, − 0.08) within the medium tercile compared to the same estimate for the high-socioeconomic status regions. The grip strength of the individuals classified in the “several conditions” class was better for those living within the medium (1.61 points higher, 95% CI = 0.22,3.19) and the lowest (1.72, 95% CI = 0.34,3.28) socioeconomic status tercile.

Finally, we also detected some differences regarding the association between latent class membership and healthcare use across socioeconomic status terciles. The probability of being hospitalized when belonging to the “osteoarticular” class was lower within the medium socioeconomic status tercile regions compared to the high-socioeconomic status zones (0.80 times, 95% CI = 0.65–0.98). The probabilities of having 4 or more doctor visits per year within the “metabolic,” “cardiovascular” and “several conditions” classes for the lowest tercile were higher in comparison with the high-socioeconomic status regions (1.38 times higher, 95% CI = 1.23–1.52; 1.72, 95% CI = 1.18–2.55; and 1.41, 95% CI = 1.07–1.91, respectively).

The results of the sensitivity analyses using the region socioeconomic status as a continuous variable (Additional file 1 Table S7) were mostly consistent with the described findings.

## Discussion

Our study analyzed the association between several multimorbidity patterns and health-related outcomes in a 50+ European representative sample of 55,915 individuals. We identified 6 classes and found significant differences in their associated physical limitations, healthcare utilization and QoL indicators. This work also assessed how such relationships varied with the territorial socioeconomic status, yielding significant results.

Regarding our first aim, we found that individuals classified within any non-healthy class had more probabilities of worse health outcomes and a higher healthcare utilization. Among the non-heathy classes, the “metabolic” class had the least severe outcomes and the lower healthcare use. This finding is consistent with previous publications that reported similar groups with more probabilities of being limited (Bayes-Marin et al. 2020; Olaya et al. 2017), using health services (Olaya et al. 2017; Whitson et al. 2016), and having a worse QoL (Olaya et al. 2017; Larsen et al. 2017; Zheng et al. 2020) and self-reported health (Bayes-Marin et al. 2020) than the healthiest individuals, but with better outcomes when compared to further multimorbid groups.

The estimated probabilities of 4+ doctor visits and of being hospitalized for the “osteoarticular” class were similar to those obtained for the “metabolic” group, but the former showed a slightly worse self-reported health, CASP index score and grip strength. However, the physical limitation indicators suggested a further impairment and were close to the identified “cardiovascular” class, consistent with the mobility restrictions related to the musculoskeletal conditions characterizing that pattern.

The “cardiovascular” class appeared to have lesser physical limitations than the “neuro-affective-ulcer” and the “several conditions” classes and a better quality of life according to the CASP index. This finding agrees with a previous publication that reported a heart disease class having a worse QoL when compared to the vascular risk (metabolic) and healthy classes, but better in comparison with a class with several chronic conditions (Zheng et al. 2020). Despite of having better indicators regarding physical limitations and QoL, the “cardiovascular” class probabilities of reporting a bad health status were similar to those estimated for the “neuro-affective-ulcer” and the “several conditions” classes. Furthermore, these three classes had similar probabilities of 4+ doctor visits, and the “cardiovascular” class had the highest probability of hospitalization, along with the “several conditions” class. Finally, the “neuro-affective-ulcer” class had a worse self-reported health and CASP score compared to the “several conditions” class, even if the probability of being limited was similar between both groups.

Regarding the second aim of our study, we did detect some significant differences of the latent class membership associations with the analyzed outcomes across regions with different levels of socioeconomic status, even after adjusting for the individual socioeconomic status. A previous study reported that socioeconomic deprivation and multimorbidity were both associated with frailty when assessed at the same time (Hanlon et al. 2018). Our study went a step further as we explored the interaction between multimorbidity and an ecological deprivation variable when relating them to physical limitation and QoL.

The increased probability of being limited for people classified within the “metabolic” class appeared to be stronger in regions within the lowest socioeconomic status tercile, as well as the reduction of the QoL according to the CASP index and the probability of reporting a bad health status. The “osteoarticular” class membership also had a stronger association with the probability of being limited and reporting a bad health status within the lowest socioeconomic status tercile. These findings suggest that a wealthier socioeconomic situation of the region may alleviate the relationship between the metabolic and osteoarticular conditions and the limitation and QoL.

Fewer significant interactions with the region socioeconomic status were detected across the “cardiovascular,” the “neuro-affective-ulcer” and the “several conditions” classes. For the “cardiovascular” class, we only found a reduced probability of being limited according to the IADL indicator within the medium socioeconomic status tercile and an increased probability of having two or more mobility limitations within the lowest socioeconomic status tercile. The “several conditions” and the “cardiovascular” classes associations with the doctor visits appeared to be stronger in regions within the lowest socioeconomic tercile. Regarding the “several conditions” class, an increased association for the GALI limitation and a bad self-perceived health was detected within the lowest socioeconomic status tercile. However, we found a better grip strength for individuals living in regions within the medium and the lowest socioeconomic status tercile and classified within the “several conditions” class when compared to individuals within the highest tercile and belonging to the same multimorbidity group.

The results arising from the region socioeconomic status-stratified analyses suggested that living within a unwealthier region might exacerbate the association of having the chronic conditions characterizing our “metabolic” and “osteoarticular” classes with physical limitation, QoL and health perception. Nevertheless, fewer and less consistent evidence was found in this direction for multimorbidity patterns having more severe chronic conditions and worse outcomes. This fact might indicate that the environmental socioeconomic status has a smaller relevance on the effects of multimorbidity over physical limitations and QoL when having more impaired conditions. Another explanation for our results could be the smaller sample classified within the more severe multimorbidity classes, reducing the detection of significant interactions.

This is, to the best of our knowledge, the first study to analyze how a socioeconomic variable changes the association of multimorbidity patterns with health-related outcomes. The strengths of our study included a large sample size of 55,915 individuals arising from a representative cohort covering several European countries.

However, our study also has some limitations. The generalization of the results might be affected by a non-response bias, with the individual response rate of the 7th wave of SHARE ranging from 30.63% (Croatia) to 60.56% (Bulgaria) in baseline/refreshment samples and from 51.48% (Luxembourg) to 84.71% (Croatia) in longitudinal samples (Bergmann et al. 2019). The measure of chronic conditions was self-reported, thus implying a potential response bias. The analyses were also limited by the number of conditions probed during SHARE interviews, and it is worth considering that the presence of osteoporosis might be underestimated since it was assessed considering the self-reported treatment. In addition, the socioeconomic status was measured for regions covering considerable areas, resulting in a low resolution of this ecological variable. The region information was only available for the first wave in which each participant appeared in the study, ignoring possible location changes. Furthermore, the results of the interaction models could be confounded by a potential heterogeneity in multimorbidity patterns across territories, even if a sensitivity analysis showed no large differences in the prevalence rates for chronic conditions when stratified by most likely class and region socioeconomic status tercile (Additional file 1 Fig. S3). Finally, the identified classes have little relevance for patient classification within a single multimorbidity group in clinical practice due to the lack of enough class separation, with an entropy of 0.570. In this sense, our results provide evidence on multimorbidity from an epidemiological perspective, and the low class separation was accounted for by modeling the health-related outcomes with a methods taking into account latent class measurement error.

## Conclusions

This study identified and characterized six distinct multimorbidity patterns and examined their association with physical limitations, quality of life and healthcare utilization. The findings have important implications for targeting health promotion strategies. Additionally, our results showed disparities between regions, revealing that multimorbidity might have more profound consequences on individuals’ lives in regions with lower GDP. These results offer valuable insights into the patterns of chronic diseases that are more prone to contribute to health disparities and highlight the importance of addressing health inequities and working toward a more equitable healthcare system across regions.

### Supplementary Information

**Additional file 1.** Supplementary Materials.

## Data Availability

The original dataset analyzed in this publication is publicly available at the SHARE project website after registration. The scripts containing the code used to carry out our analyses (including the database assembling and data modeling) can be found at https://doi.org/10.5281/zenodo.5704883.
