# Supplementary material for: Multimorbidity patterns and disability and healthcare use in Europe: do the associations change with the regional socioeconomic status?
Source: Eur J Ageing. 2024 Jan 3;21(1):1. doi: 10.1007/s10433-023-00795-6 (PMC10764705; doi:10.1007/s10433-023-00795-6)
Supplement: Supplementary file 1 — Additional file 1. Supplementary Materials. [file 10433_2023_795_MOESM1_ESM.docx]

**Supplementary Materials. Multimorbidity patterns and their health-related outcomes in Europe: Does the region** **socioeconomic status mediate the impact of comorbidities on disability and healthcare use?**

**Supplementary Methods 1.** NUTS conversion

**Supplementary Figure 1.** Mediation analysis conceptualization

**Supplementary Methods 2.** Mediation analysis specification

**Supplementary Figure 2.** Sample flowchart

**Supplementary Table 1.** Basic descriptive statistics by gender and region socioeconomic status tercile

**Supplementary Results.** Selection of the optimal latent class number and entropy

**Supplementary Table 2**. Latent Class Analysis Fit Statistics for latent class number selection

**Supplementary Table 3.** Latent class-specific probabilities for each chronic disease resulting from the main analyses

**Supplementary Table 4.** Latent class-specific probabilities for each chronic disease resulting from the sensitivity analysis using country weights

**Supplementary Table 5.** Odds ratios and mean differences for the unadjusted models assessing the association between latent class membership and the health-related outcomes

**Supplementary Table 6.** Odds ratios and adjusted mean differences for the adjusted models assessing the association between latent class membership and the health-related outcomes

**Supplementary Table 7.** Influence of the region socioeconomic status (expressed as a continuous variable) on the effect that each multimorbidity class membership has on each analyzed outcome

**Supplementary Figure 3.** Class-specific disease prevalence by most likely class and stratified by NUTS 2 socioeconomic status tercile

**Supplementary Methods 1. NUTS conversion**

The NUTS regional classification system has been constantly changing. Since the SHARE study registered the NUTS region only for the first wave each participant appeared in, we had to converse the regional codes assigned to each individual from one version to the subsequent from 2003 to 2016.

To that end, we used the conversion tables available at Eurostat.^1^ The NUTS classification had a drastic change within Denmark from the 2003 to the 2006 version, making impossible the conversion between them. The participants from that country whose region was registered with the 2003 version (those sampled within the 1^st^ and the 2^nd^ wave) were thus excluded.

Another drastic change between versions (concerning the 2^nd^ NUTS level) led to assess the *PL9* Polish region using the 1^st^ NUTS level. Since the socioeconomic status indicator (the Gross Domestic Product (GDP) in purchasing power (PPS) per inhabitant)^2^ was only available by NUTS 2 regions, we computed a population-weighted mean of this indicator of the two NUTS 2 composing the *PL9* region.

In addition, the NUTS regions of SHARE participants from Germany were only available at the 1^st^ level. Therefore, a population-weighted mean of this indicator of the NUTS 2 regions composing each NUTS 1 region was also computed.

1 History of NUTS - NUTS - Nomenclature of territorial units for statistics - Eurostat. https://ec.europa.eu/eurostat/web/nuts/history (accessed Aug 17, 2021).

2 European Commission. Regional gross domestic product (PPS per inhabitant) by NUTS 2 regions. Eurostat. 2017. https://ec.europa.eu/eurostat/web/products-datasets/-/tgs00005 (accessed July 6, 2021).

**Supplementary Figure 1.** Mediation analysis conceptualization

Multimorbidity classes are estimated (from the presence/absence of several chronic conditions) as a latent (non-observed) variable. Multimorbidity classes are then analyzed as a predictor of each outcome (including adjusting covariates). In mediation analyses, the region’s socioeconomic status tercile is included as a possible modulator of the effect of multimorbidity classes over each outcome.

...

NUTS 2 socioeconomic status tercile

Adjusting covariates (sex, age, etc.)

Outcome

Chronic condition 16

Chronic condition 3

Chronic condition 2

Chronic condition 1

**Supplementary Methods 2. Mediation analysis specification**

The proposed mediation analysis aimed to assess whether the region socioeconomic status tercile modifies the effect of belonging to a certain multimorbidity class on the probability of having an outcome (for categorical variables) or on the mean value of the outcome (for continuous variables). The BCH method was the chosen approach for including distal outcomes and adjust for covariates using auxiliary models.

In the mediation analysis, the NUTS region socioeconomic status was assessed as a categorical variable with three levels (divided in terciles), included using two dummy variables (necessary within the Mplus framework). The auxiliary models were implemented as represented in the following figure, allowing the effect of the NUTS region socioeconomic status tercile to vary between latent classes:

Outcome

Adjusting covariates

NUTS 2 socioeconomic status tercile

**Categorical binary outcomes**

For categorical outcomes, we obtained class-specific regression coefficients for the effect of living in a region within the medium or highest socioeconomic status terciles (taking the lowest as the baseline) on the probability of having that outcome, along with class-specific intercepts. Therefore, the odds of having an event (for example, being disabled) for the class *k* and the *j^th^* socioeconomic status tercile is computed as follows:

$$\log\left( \frac{P}{1-P} \right)=\alpha_{k}+\beta_{kj}+\beta_{age}*x_{age}+\ldots$$

With this information, for each socioeconomic status tercile *j*, one may calculate the odds ratio of having the event for every *k*-1 multimorbidity classes *i,* using the healthiest class as a reference:

$${Odds ratio}_{ij}= \frac{\frac{P_{ij}}{1-P_{ij}}}{\frac{P_{healthy j}}{1-P_{healthy j}}}= \frac{exp(\alpha_{i}+\beta_{ij})}{exp(\alpha_{healthy}+\beta_{healthy, j})}$$

where *P_healthy, j_* is the probability of having the event for an individual belonging to the healthy class and living within the *j^th^* tercile and *P_i j_* is the probability of having the event for an individual belonging to the *I^th^* class and living within the *j^th^* tercile.

One may then compare the odds ratio for the *i*^th^ class between two socioeconomic status terciles as follows:

$$\frac{{Odds ratio}_{i,j}}{{Odds ratio}_{i,j+1}}$$

If the result of this ratio is significantly higher than 1, one may affirm that the association between belonging to the *i^th^* multimorbidity class on having the event in greater in the *j^th^* when compared to the *j^th^+1* tercile, and smaller if the value is significantly smaller than 1. Otherwise, there would not be a difference in the effect, and no mediation role of the region’s socioeconomic status would be mediating the relationship between that multimorbidity class and the probability of having an outcome.

**Continuous outcomes**

For continuous outcomes, we obtained class-specific regression coefficients for the effect of living in a region within the medium or highest socioeconomic status terciles (taking the lowest as the baseline) on the estimated outcome, along with class-specific intercepts. Therefore, the estimated mean for a continuous outcome for the class *k* and the *j^th^* socioeconomic status tercile is computed as follows:

$$Y=\alpha_{k}+\beta_{kj}+\beta_{age}*x_{age}+\ldots$$

With this information, for each socioeconomic status tercile *j*, one may calculate the adjusted mean difference of the continuous outcome for every *k*-1 multimorbidity classes *i,* using the healthiest class as a reference:

$${Adjusted mean difference}_{ij}= Y_{healthy, j}-Y_{ij}$$

where *Y_healthy, j_* is the estimated outcome value for an individual belonging to the healthy class and living within the *j^th^* tercile and *Y_i j_* is the same estimate for an individual belonging to the *I^th^* class and living within the *j^th^* tercile.

One may then compare the adjusted mean difference for the *i*^th^ class between two socioeconomic status terciles as follows:

$${Adjusted mean diference}_{i,j}-{Adjusted mean difference}_{i,j+1}$$

If the result of this difference is significantly higher than 0, one may affirm that the association between belonging to the *i^th^* multimorbidity class on the estimated outcome is “more positive” in the *j^th^* when compared to the *j^th^+1* tercile, and “more negative” if the value is significantly smaller than 0. Otherwise, there would not be a difference in the effect, and no mediation role of the region’s socioeconomic status would be mediating the relationship between that multimorbidity class and the estimated continuous outcome.

**Supplementary Figure 2.** Sample flowchart with exclusion criteria

Individuals interviewed during the 7^th^ SHARE wave

N = 77,263

Individuals living within Israel or part of the Girona’s Country Team sample

N = 4,273

Final analyzed sample

N = 55,915

Individuals living within Switzerland

N = 2,402

Individuals aged less than 50 years old

N = 872

Individuals from Denmark with NUTS registered with the 2003 version

N = 1,393

Individuals with missing information for the NUTS region

N = 692

Individuals with missing information for the variables included within the models

N = 11,716

| Supplementary Table 1. Basic descriptive statistics by gender and region socioeconomic status tercile. | | | | | | |
| --- | --- | --- | --- | --- | --- | --- |
|  | Total | Sex | | Region socioeconomic status tercile | | |
|  |  | Males | Females | Lowest | Medium | Highest |
| N | 55,915 | 24,738 | 31,177 | 18,907 | 18,483 | 18,525 |
| Age | 67.24 (9.28) | 67.52 (9.10) | 67.02 (9.42) | 66.08 (9.21) | 67.83 (9.31) | 67.85 (9.23) |
| BMI Under | 564 (0.01) | 122 (0.00) | 442 (0.01) | 166 (0.01) | 164 (0.01) | 234 (0.01) |
| BMI Normal | 17,945 (0.32) | 6,955 (0.28) | 10,990 (0.35) | 5,288 (0.28) | 5,602 (0.30) | 7,055 (0.38) |
| BMI Overweight | 23,517 (0.42) | 11,760 (0.48) | 11,757 (0.38) | 8,215 (0.43) | 7,749 (0.42) | 7,553 (0.41) |
| BMI Obese | 13,889 (0.25) | 5,901 (0.24) | 7,988 (0.26) | 5,238 (0.28) | 4,968 (0.27) | 3,683 (0.20) |
| Pre-secondary education | 18,892 (0.34) | 7,500 (0.30) | 11,392 (0.37) | 6,726 (0.36) | 6,771 (0.37) | 5,395 (0.29) |
| Secondary education | 21,830 (0.39) | 10,328 (0.42) | 11,502 (0.37) | 8,432 (0.45) | 6,572 (0.36) | 6,826 (0.37) |
| Post-secondary education | 15,193 (0.27) | 6,910 (0.28) | 8,283 (0.27) | 3,749 (0.20) | 5,140 (0.28) | 6,304 (0.34) |
| Great difficulty | 6,822 (0.12) | 2,706 (0.11) | 4,116 (0.13) | 4,059 (0.21) | 1,869 (0.10) | 894 (0.05) |
| Some difficulty | 16,731 (0.30) | 7,035 (0.28) | 9,696 (0.31) | 8,104 (0.43) | 5,264 (0.28) | 3,363 (0.18) |
| Fairly easily | 16,900 (0.30) | 7,617 (0.31) | 9,283 (0.30) | 4,814 (0.25) | 6,276 (0.34) | 5,810 (0.31) |
| Easily | 15,462 (0.28) | 7,380 (0.30) | 8,082 (0.26) | 1,930 (0.10) | 5,074 (0.27) | 8,458 (0.46) |
| Activity Daily Limitations (1 or more) | 5,378 (0.10) | 2,263 (0.09) | 3,115 (0.10) | 2,051 (0.11) | 1,864 (0.10) | 1,463 (0.08) |
| Instrumental Activity Daily Limitations (1 or more) | 8,524 (0.15) | 2,943 (0.12) | 5,581 (0.18) | 3,095 (0.16) | 3,020 (0.16) | 2,409 (0.13) |
| GALI limitations (with limitations) | 25,918 (0.46) | 10,897 (0.44) | 15,021 (0.48) | 8,929 (0.47) | 8,964 (0.48) | 8,025 (0.43) |
| Mobility Limitations (2 or more) | 27,541 (0.49) | 10,460 (0.42) | 17,081 (0.55) | 10,077 (0.53) | 9,236 (0.50) | 8,228 (0.44) |
| Self-perceived health (fair or poor) | 44,098 (0.79) | 19,212 (0.78) | 24,886 (0.80) | 15,528 (0.82) | 15,123 (0.82) | 13,447 (0.73) |
| Hospitalization (yes) | 7,745 (0.14) | 3,698 (0.15) | 4,047 (0.13) | 2,451 (0.13) | 2,530 (0.14) | 2,764 (0.15) |
| doctor visits (4+) | 29,851 (0.54) | 12,576 (0.51) | 17,275 (0.55) | 9,558 (0.51) | 10,098 (0.55) | 10,195 (0.55) |
| CASP index | 36.91 (6.33) | 37.38 (6.16) | 36.55 (6.44) | 35.09 (6.56) | 36.82 (6.03) | 38.88 (5.79) |
| Grip Stength | 33.01 (11.57) | 41.87 (10.15) | 25.98 (6.83) | 32.27 (11.58) | 32.69 (11.42) | 34.09 (11.61) |
| Cardiovascular disease | 6,698 (0.12) | 3,657 (0.15) | 3,041 (0.10) | 2,397 (0.13) | 2,450 (0.13) | 1,851 (0.10) |
| Hypertension | 25,001 (0.45) | 10,810 (0.44) | 14,191 (0.46) | 8,998 (0.48) | 8,468 (0.46) | 7,535 (0.41) |
| High blood cholesterol | 14,036 (0.25) | 5,951 (0.24) | 8,085 (0.26) | 4,254 (0.22) | 5,141 (0.28) | 4,641 (0.25) |
| Cerebro-vascular disease | 2,088 (0.04) | 1,097 (0.04) | 991 (0.03) | 823 (0.04) | 661 (0.04) | 604 (0.03) |
| Diabetes | 7,463 (0.13) | 3,675 (0.15) | 3,788 (0.12) | 2,550 (0.13) | 2,791 (0.15) | 2,122 (0.11) |
| COPD | 2,984 (0.05) | 1,413 (0.06) | 1,571 (0.05) | 877 (0.05) | 1,053 (0.06) | 1,054 (0.06) |
| Osteoarthritis | 10,738 (0.19) | 3,413 (0.14) | 7,325 (0.23) | 2,954 (0.16) | 4,298 (0.23) | 3,486 (0.19) |
| Rheumatoid arthritis | 5,605 (0.10) | 1,753 (0.07) | 3,852 (0.12) | 2,193 (0.12) | 1,836 (0.10) | 1,576 (0.09) |
| Osteoporosis | 10,738 (0.19) | 3,413 (0.14) | 7,325 (0.23) | 2,954 (0.16) | 4,298 (0.23) | 3,486 (0.19) |
| Affective disorders | 3,515 (0.06) | 971 (0.04) | 2,544 (0.08) | 1,153 (0.06) | 1,296 (0.07) | 1,066 (0.06) |
| Cancer | 2,599 (0.05) | 1,246 (0.05) | 1,353 (0.04) | 762 (0.04) | 941 (0.05) | 896 (0.05) |
| Ulcer | 2,430 (0.04) | 1,027 (0.04) | 1,403 (0.05) | 1,070 (0.06) | 811 (0.04) | 549 (0.03) |
| Parkinson Disease | 366 (0.01) | 208 (0.01) | 158 (0.01) | 118 (0.01) | 131 (0.01) | 117 (0.01) |
| Cataracts | 4,518 (0.08) | 1,750 (0.07) | 2,768 (0.09) | 1,256 (0.07) | 1,663 (0.09) | 1,599 (0.09) |
| Alzheimer's Disease | 655 (0.01) | 302 (0.01) | 353 (0.01) | 227 (0.01) | 192 (0.01) | 236 (0.01) |
| Kidney Disease | 1,283 (0.02) | 552 (0.02) | 731 (0.02) | 568 (0.03) | 407 (0.02) | 308 (0.02) |
|  | | | | | | |

**Supplementary Results. Selection of the optimal latent class number**

The fit statistics used to select the best class number are displayed in Supplementary Table 2, along with the entropy of each model. There was not a clear optimal solution. The BIC pointed to a 5-class solution whereas the aBIC suggested a 7-class solution. On the other hand, the LMR test suggested that the 7-class solution did not have a better fit than the 6-class solution, but the same test indicated that the 8-class solution had a better fit than the 7-class model. The final chosen solution was the model with 6 different classes, since it maintained a compromise between the BIC and the aBIC (pointing to a 5-class and a 7-class solution respectively) and because the LMR test pointed to that model when compared to the 5-class and the 7-class solutions.

| **Supplementary Table 2**. Latent Class Analysis Fit Statistics for latent class number selection and entropy | | | | | |
| --- | --- | --- | --- | --- | --- |
| **Number of classes** | **AIC** | **BIC** | **aBIC** | **LMR test p-value** | **Entropy** |
| 1 | 502237,468 | 502380,374 | 502329,525 |  |  |
| 2 | 486839,841 | 487134,584 | 487029,709 | <0.001 | 0,521 |
| 3 | 484137,089 | 484583,669 | 484424,768 | <0.001 | 0,555 |
| 4 | 482760,931 | 483359,347 | 483146,420 | <0.001 | 0,541 |
| 5 | 482401,31 | **483151,564** | 482884,610 | <0.001 | 0,559 |
| 6 | 482257,803 | 483159,893 | 482838,913 | **0,007** | 0,570 |
| 7 | 482140,088 | 483194,015 | **482819,009** | 0,2429 | 0,538 |
| 8 | **482059,968** | 483265,732 | 482836,700 | **0,0016** | **0,576** |
| *Note*: Akaike Information Criterion (AIC), Bayesian Information Criterion (BIC), Sample-Size Adjusted BIC (aBIC), Lo-Mendell-Rubin Likelihood Ratio test (LMR). The optimal fit values are displayed in bold. | | | | | |

| **Supplementary Table 3.** Latent class-specific probabilities for each chronic disease resulting from the main analyses | | | | | | |
| --- | --- | --- | --- | --- | --- | --- |
| **Chronic condition** | **Several conditions** | **Neuro-affective-ulcer** | **Cardiovascular** | **Osteoarticular** | **Metabolic** | **Healthy** |
| **Heart disease** | 0.392 (0.349-0.435) | 0.228 (0.143-0.312) | 0.445 (0.329-0.56) | 0.081 (0.064-0.098) | 0.161 (0.141-0.182) | 0.045 (0.039-0.051) |
| **Hypertension** | 0.904 (0.872-0.936) | 0.432 (0.337-0.528) | 0.605 (0.505-0.705) | 0.418 (0.381-0.455) | 0.835 (0.803-0.868) | 0.218 (0.201-0.234) |
| **High blood cholesterol** | 0.721 (0.677-0.766) | 0.195 (0.113-0.277) | 0.352 (0.211-0.493) | 0.261 (0.232-0.291) | 0.498 (0.455-0.54) | 0.087 (0.079-0.095) |
| **Cerebrovascular disease** | 0.185 (0.157-0.213) | 0.123 (0.067-0.178) | 0.131 (0.082-0.18) | 0.01 (0.003-0.017) | 0.048 (0.039-0.058) | 0.009 (0.008-0.011) |
| **Diabetes** | 0.426 (0.384-0.469) | 0.057 (0.011-0.102) | 0.33 (0.216-0.444) | 0.064 (0.048-0.081) | 0.281 (0.258-0.304) | 0.04 (0.035-0.044) |
| **COPD** | 0.219 (0.188-0.25) | 0.193 (0.148-0.239) | 0.135 (0.083-0.187) | 0.079 (0.064-0.094) | 0.034 (0.026-0.042) | 0.024 (0.021-0.027) |
| **Osteoarthritis** | 0.547 (0.5-0.594) | 0.235 (0.135-0.335) | 0.172 (0.104-0.24) | 0.659 (0.6-0.717) | 0.158 (0.139-0.177) | 0.079 (0.071-0.088) |
| **Rheumatoid arthritis** | 0.392 (0.349-0.435) | 0.257 (0.207-0.306) | 0.061 (-0.007-0.128) | 0.251 (0.226-0.276) | 0.108 (0.096-0.12) | 0.032 (0.028-0.036) |
| **Osteoporosis** | 0.217 (0.182-0.253) | 0.082 (0.03-0.134) | 0* | 0.279 (0.246-0.313) | 0.014 (0.007-0.02) | 0.011 (0.008-0.013) |
| **Affective disorder** | 0.28 (0.243-0.316) | 0.318 (0.182-0.455) | 0* | 0.093 (0.072-0.114) | 0.051 (0.041-0.061) | 0.03 (0.027-0.034) |
| **Cancer** | 0.122 (0.1-0.144) | 0.089 (0.06-0.118) | 0.141 (0.081-0.202) | 0.054 (0.043-0.065) | 0.036 (0.028-0.044) | 0.031 (0.028-0.034) |
| **Peptic ulcer** | 0.204 (0.174-0.234) | 0.185 (0.13-0.241) | 0.038 (0.008-0.068) | 0.07 (0.054-0.085) | 0.037 (0.03-0.044) | 0.017 (0.014-0.02) |
| **Parkinson's disease** | 0.025 (0.016-0.034) | 0.037 (0.017-0.058) | 0.016 (0-0.032) | 0.004 (0.001-0.008) | 0.004 (0.001-0.006) | 0.004 (0.003-0.004) |
| **Cataracts** | 0.332 (0.3-0.365) | 0.176 (0.132-0.22) | 0.205 (0.125-0.285) | 0.148 (0.13-0.165) | 0.08 (0.066-0.093) | 0.026 (0.023-0.03) |
| **Dementia** | 0.059 (0.044-0.075) | 0.114 (0.064-0.165) | 0.024 (0.001-0.047) | 0.001 (0-0.005) | 0.005 (0.001-0.008) | 0.004 (0.003-0.005) |
| **Chronic kidney disease** | 0.165 (0.139-0.191) | 0.055 (0.028-0.083) | 0.092 (0.054-0.13) | 0.019 (0.012-0.026) | 0.016 (0.01-0.022) | 0.006 (0.004-0.007) |
| *These parameters were fixed during the estimation process | | | | | |  |

| **Supplementary Table 4.** Latent class-specific probabilities for each chronic disease resulting from the sensitivity analysis using country weights | | | | | | |
| --- | --- | --- | --- | --- | --- | --- |
| **Chronic condition** | **Several conditions** | **Neuro-affective-ulcer** | **Cardiovascular** | **Osteoarticular** | **Metabolic** | **Healthy** |
| **Heart disease** | 0.416 (0.347-0.485) | 0.244 (0.133-0.354) | 0.487 (0.344-0.631) | 0.09 (0.065-0.114) | 0.148 (0.12-0.177) | 0.047 (0.039-0.055) |
| **Hypertension** | 0.907 (0.85-0.964) | 0.374 (0.23-0.517) | 0.546 (0.379-0.713) | 0.433 (0.378-0.489) | 0.83 (0.781-0.879) | 0.212 (0.19-0.233) |
| **High blood cholesterol** | 0.737 (0.676-0.798) | 0.137 (0.007-0.266) | 0.374 (0.217-0.532) | 0.269 (0.225-0.313) | 0.491 (0.446-0.535) | 0.087 (0.075-0.099) |
| **Cerebrovascular disease** | 0.197 (0.152-0.243) | 0.13 (0.027-0.233) | 0.108 (0.058-0.158) | 0.006 (0-0.016) | 0.044 (0.032-0.057) | 0.01 (0.007-0.013) |
| **Diabetes** | 0.444 (0.373-0.514) | 0.065 (0.005-0.125) | 0.349 (0.249-0.45) | 0.066 (0.042-0.091) | 0.3 (0.27-0.33) | 0.043 (0.036-0.051) |
| **COPD** | 0.225 (0.178-0.273) | 0.201 (0.121-0.282) | 0.142 (0.078-0.206) | 0.092 (0.069-0.115) | 0.039 (0.027-0.051) | 0.023 (0.019-0.028) |
| **Osteoarthritis** | 0.541 (0.475-0.607) | 0.207 (0.067-0.346) | 0.282 (0.189-0.376) | 0.667 (0.571-0.762) | 0.192 (0.163-0.222) | 0.104 (0.091-0.117) |
| **Rheumatoid arthritis** | 0.467 (0.391-0.543) | 0.241 (0.136-0.346) | 0.009 (-0.07-0.088) | 0.282 (0.243-0.321) | 0.115 (0.094-0.136) | 0.03 (0.024-0.037) |
| **Osteoporosis** | 0.182 (0.13-0.234) | 0.076 (0-0.153) | 0* | 0.249 (0.204-0.294) | 0.016 (0.007-0.024) | 0.012 (0.008-0.017) |
| **Affective disorder** | 0.297 (0.235-0.358) | 0.312 (0.146-0.478) | 0* | 0.133 (0.105-0.161) | 0.065 (0.049-0.08) | 0.033 (0.027-0.039) |
| **Cancer** | 0.129 (0.095-0.163) | 0.131 (0.077-0.185) | 0.155 (0.095-0.216) | 0.05 (0.034-0.066) | 0.033 (0.022-0.045) | 0.032 (0.027-0.037) |
| **Peptic ulcer** | 0.181 (0.133-0.228) | 0.082 (0.006-0.158) | 0.055 (0.018-0.092) | 0.068 (0.051-0.084) | 0.031 (0.023-0.038) | 0.012 (0.01-0.015) |
| **Parkinson's disease** | 0.033 (0.016-0.051) | 0.054 (0.012-0.097) | 0.013 (-0.004-0.029) | 0.003 (-0.002-0.009) | 0.006 (0.002-0.009) | 0.004 (0.003-0.006) |
| **Cataracts** | 0.345 (0.294-0.397) | 0.2 (0.118-0.282) | 0.206 (0.124-0.289) | 0.144 (0.12-0.168) | 0.092 (0.074-0.11) | 0.025 (0.02-0.03) |
| **Dementia** | 0.078 (0.044-0.111) | 0.149 (0.035-0.263) | 0* | 0* | 0.007 (0.002-0.011) | 0.004 (0.002-0.007) |
| **Chronic kidney disease** | 0.184 (0.141-0.227) | 0.035 (0.005-0.065) | 0.102 (0.059-0.146) | 0.023 (0.013-0.034) | 0.012 (0.004-0.021) | 0.006 (0.004-0.008) |
| *These parameters were fixed during the estimation process | | | | | |  |

| **Supplementary Table 5.** Odds ratios and mean differences for the unadjusted models assessing the association between latent class membership and the health-related outcomes | | | | | |
| --- | --- | --- | --- | --- | --- |
| variable | **Several conditions** | **Neuro-affective-ulcer** | **Cardiovascular** | **Osteoarticular** | **Metabolic** |
| **ADL (1+)^1^** | 32.382 (26.393, 40.031) | 33.442 (24.619, 45.411) | 15.629 (10.960, 21.956) | 9.341 (7.396, 11.644) | 3.963 (3.071, 5.206) |
| **IADL (1+)^1^** | 28.732 (30.672, 57.838) | 41.262 (30.672, 57.838) | 14.777 (11.078, 20.208) | 8.191 (6.988, 9.810) | 3.480 (2.895, 4.155) |
| **GALI limitations^1^** | 42.891 (31.633, 63.433) | 146.055 (44.715, -)^a^ | 194.150 (43.619, -)^a^ | 12.303 (10.610, 14.148) | 3.578 (3.287, 3.930) |
| **Mobility limitations (2+)^1^** | 94.845 (56.617, 358.858) | 30.113 (18.658, 67.309) | 23.707 (15.403, 45.363) | 17.477 (14.683, 21.005) | 4.520 (4.150, 4.964) |
| **Self-perceived health (bad)^1^** | 34.951 (3.595, 66.308) | -^b^ | -^b^ | 10.280 (7.349, 13.211) | 7.265 (6.196, 8.333) |
| **Hospitalization (Yes)^1^** | 7.793 (6.710, 8.922) | 4.648 (3.452, 6.222) | 14.524 (11.120, 18.421) | 2.531 (2.117, 2.933) | 1.892 (1.599, 2.185) |
| **Doctor visits (4+)^2^** | 26.141 (20.005, 36.430) | 11.350 (8.169, 17.072) | 98.420 (29.848, -) ^a^ | 7.904 (6.913, 9.072) | 6.276 (5.747, 6.870) |
| **CASP index^2^** | -8.126 (-8.576, -7.712) | -9.216 (-10.082, -8.356) | -4.675 (-5.499, -3.879) | -3.506 (-3.839, -3.192) | -2.443 (-2.687, -2.196) |
| **Grip strength^2^** | -11.673 (-12.420, -10.899) | -9.300 (-10.665, -7.823) | -2.642 (-4.092, -1.244) | -10.106 (-10.727, -9.547) | -2.985 (-3.484, -2.548) |
| *Note*: 95% confidence intervals are displayed.  ^a^These upper confidence limits were not available when estimating with bootstrap, since some parameters were fixed during some replications.  ^b^These odds ratios could not be computed since the probabilities of having a bad self-perceived health for these classes were fixed to 1 during estimation.  ^1^Categorical outcomes; odds ratio  ^2^Continuous outcomes; mean differences | | | | | |

| **Supplementary Table 6.** Odds ratios and adjusted mean differences for the adjusted models assessing the association between latent class membership and the health-related outcomes | | | | | |
| --- | --- | --- | --- | --- | --- |
| variable | **Several conditions** | **Neuro-affective-ulcer** | **Cardiovascular** | **Osteoarticular** | **Metabolic** |
| **ADL (1+)^1^** | 5.103 (4.560, 5.546) | 5.598 (4.747, 6.501) | 3.092 (2.661, 3.581) | 2.853 (2.586, 3.111) | 1.681 (1.558, 1.793) |
| **IADL (1+)^1^** | 4.750 (5.420, 7.237) | 6.306 (5.420, 7.237) | 3.220 (2.810, 3.688) | 2.520 (2.330, 2.724) | 1.629 (1.533, 1.716) |
| **GALI limitations^1^** | 7.427 (6.624, 8.234) | 8.354 (7.012, 9.973) | 6.012 (5.262, 6.964) | 4.296 (3.999, 4.592) | 2.161 (2.071, 2.252) |
| **Mobility limitations (2+)^1^** | 7.387 (6.611, 8.387) | 5.913 (4.967, 7.072) | 4.319 (3.752, 4.955) | 4.526 (4.217, 4.863) | 2.177 (2.084, 2.284) |
| **Self-perceived health (bad)^1^** | 6.988 (5.833, 8.192) | 13.925 (9.057, 23.324) | 11.006 (7.951, 16.166) | 3.740 (3.382, 4.194) | 3.033 (2.852, 3.221) |
| **Hospitalization (Yes)^1^** | 3.747 (3.393, 4.119) | 2.683 (2.297, 3.130) | 4.119 (3.659, 4.642) | 1.824 (1.673, 1.981) | 1.725 (1.621, 1.825) |
| **Doctor visits (4+)^2^** | 6.748 (6.071, 7.537) | 4.509 (3.818, 5.252) | 6.053 (5.221, 7.245) | 3.355 (3.109, 3.586) | 3.068 (2.945, 3.205) |
| **CASP index^2^** | -5.84 (-6.24, -5.44) | -7.32 (-8.11, -6.62) | -3.91 (-4.65, -3.12) | -2.72 (-3.04, -2.42) | -1.52 (-1.76, -1.28) |
| **Grip strength^2^** | -4.42 (-4.93, -3.85) | -5.58 (-6.75, -4.73) | -3.38 (-4.48, -2.40) | -1.98 (-2.40, -1.58) | -0.99 (-1.31, -0.68) |
| *Note*: 95% confidence intervals are displayed. Models were adjusted for sex, age, BMI, education level, ease to make ends needs and regional socioeconomic status tercile.  ^1^Categorical outcomes; odds ratio  ^2^Continuous outcomes; adjusted mean differences | | | | | |

| **Supplementary Table 7.** Influence of the region socioeconomic status (expressed as a continuous variable) on the effect that each multimorbidity class membership has on each analyzed outcome | | | | | |
| --- | --- | --- | --- | --- | --- |
| variable | **Several conditions** | **Neuro-affective-ulcer** | **Cardiovascular** | **Osteoarticular** | **Metabolic** |
| **ADL (1+)^1^** | 0.998 (0.973, 1.023) | 1.018 (0.979, 1.061) | 1.013 (0.974, 1.057) | 1.000 (0.975, 1.025) | 1.011 (0.991, 1.032) |
| **IADL (1+)^1^** | 1.019 (0.994, 1.044) | 1.004 (0.966, 1.040) | 0.999 (0.967, 1.037) | 1.018 (0.997, 1.042) | 1.017 (1.000, 1.036)* |
| **GALI limitations^1^** | 1.023 (0.994, 1.049) | 1.007 (0.959, 1.049) | 1.040 (1.002, 1.074)* | 1.023 (1.005, 1.041)* | 1.046 (1.035, 1.057)* |
| **Mobility limitations (2+)^1^** | 1.019 (0.993, 1.043) | 1.055 (1.010, 1.095)* | 1.040 (1.008, 1.078)* | 1.033 (1.016, 1.053)* | 1.034 (1.023, 1.046)* |
| **Self-perceived health (bad)^1^** | 1.004 (0.977, 1.035) | 0.999 (0.853, 1.089) | 1.039 (0.939, 1.107) | 1.021 (0.998, 1.041) | 1.033 (1.020, 1.047)* |
| **Hospitalization (Yes)^1^** | 1.017 (0.995, 1.042) | 1.003 (0.969, 1.040) | 1.007 (0.975, 1.041) | 1.002 (0.984, 1.023) | 1.010 (0.996, 1.023) |
| **Doctor visits (4+)^2^** | 1.030 (1.002, 1.057)* | 0.998 (0.957, 1.042) | 1.041 (1.000, 1.081)* | 1.017 (0.998, 1.033) | 1.032 (1.021, 1.043)* |
| **CASP index^2^** | 0.050 (-0.052, 0.146) | -0.161 (-0.306, 0.012) | -0.120 (-0.358, 0.110) | -0.059 (-0.133, 0.013) | -0.133 (-0.202, -0.072)* |
| **Grip strength^2^** | 0.096 (-0.028, 0.234) | -0.178 (-0.371, 0.036) | -0.059 (-0.382, 0.241) | 0.021 (-0.071, 0.105) | 0.023 (-0.060, 0.111) |
| *Note*: 95% confidence intervals are displayed. Models were estimated using the region GDP (in PPS per inhabitant) included with a 10-points decrease. That is, the estimates displayed in this table show the difference of the effects of multimorbidity class memberships with a 10-points decrease in region GDP. Outcomes were modelled adjusting for sex, age, BMI, education level and ease to make ends needs. Asterisk indicates that the estimate is significant with a confidence level of 95%.  ^1^Categorical outcomes; ratio of odds ratio  ^2^Continuous outcomes; difference between adjusted mean differences | | | | | |


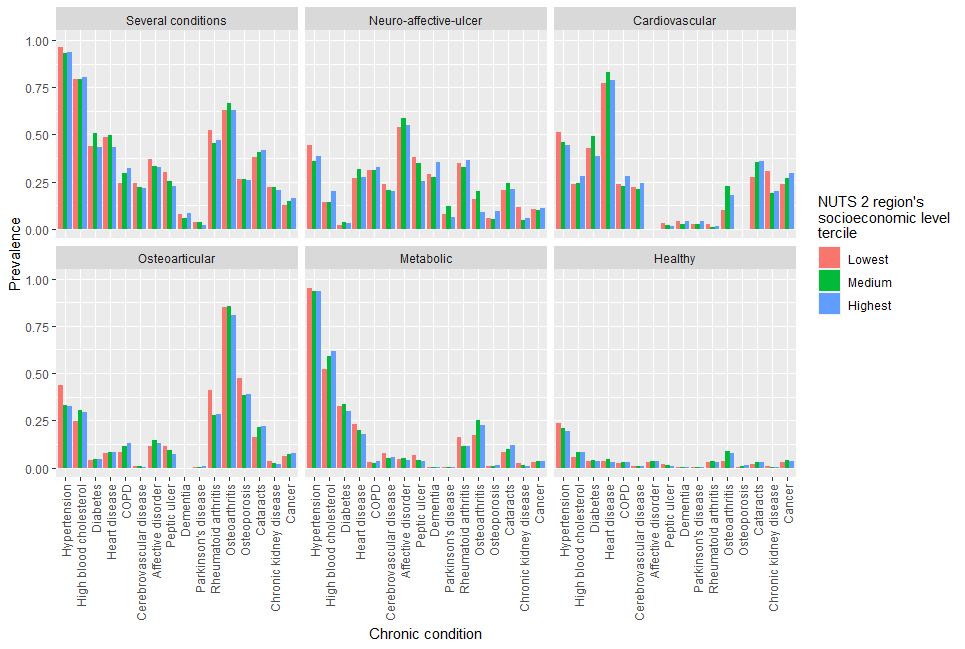


**Supplementary Figure 3.** Class-specific disease prevalence by most likely class and stratified by NUTS 2 socioeconomic status tercile.
